# Breast density in women with premature ovarian failure or postmenopausal women using hormone therapy: analytical cross-sectional study

**DOI:** 10.1590/S1516-31802010000400007

**Published:** 2010-07-01

**Authors:** Patrícia Magda Soares, César Cabello, Luis Alberto Magna, Eduardo Tinois, Cristina Laguna Benetti-Pinto

**Affiliations:** I MD, Postgraduate student, Department of Obstetrics and Gynecology, School of Medical Sciences, Universidade Estadual de São Paulo (Unicamp), Campinas, São Paulo, Brazil.; II MD, PhD. Associate professor, Department of Obstetrics and Gynecology, School of Medical Sciences, Universidade Estadual de Campinas (Unicamp), Campinas, São Paulo, Brazil.; III MD, PhD. Titular professor, Department of Medical Genetics, School of Medical Sciences, Universidade Estadual de Campinas (Unicamp), Campinas, São Paulo, Brazil.; IV Physicist and engineer, Biomedical Engineering Center, Universidade Estadual de Campinas (Unicamp), Campinas, São Paulo, Brazil.; VI MD, PhD. Professor, Department of Obstetrics and Gynecology, School of Medical Sciences, Universidade Estadual de Campinas (Unicamp), Campinas, São Paulo, Brazil.

**Keywords:** Hormone replacement therapy, Mammography, Menopause, Premature ovarian failure, Breast, Terapia de reposição hormonal, Mamografia, Menopausa, Falência ovariana prematura, Mama

## Abstract

**CONTEXT AND OBJECTIVE:**

Studies on postmenopausal women have reported increased risk of breast cancer relating to the type and duration of hormone therapy (HT) used. Women with premature ovarian failure (POF) represent a challenge, since they require prolonged HT. Little is known about the impact of prolonged HT use on these women's breasts. This study aimed to evaluate the effects of one type of HT on the breast density of women with POF, compared with postmenopausal women.

**DESIGN AND SETTING:**

Cross-sectional study at the Department of Obstetrics and Gynecology, Universidade Estadual de Campinas (Unicamp).

**METHODS:**

31 women with POF and 31 postmenopausal women, all using HT consisting of conjugated equine estrogen combined with medroxyprogesterone acetate, and matched according to HT duration, were studied. Mammography was performed on all subjects and was analyzed by means of digitization or Wolfe's classification, stratified into two categories: non-dense (N1 and P1 patterns) and dense (P2 and Dy).

**RESULTS:**

No significant difference in breast density was found between the two groups through digitization or Wolfe's classification. From digitization, the mean breast density was 24.1% ± 14.6 and 18.1% ± 17.2 in the POF and postmenopausal groups, respectively (P = 0.15). Wolfe's classification identified dense breasts in 51.6% and 29.0%, respectively (P = 0.171).

**CONCLUSION:**

There was no difference in breast density between the women with POF and postmenopausal women, who had used HT for the same length of time. These results may help towards compliance with HT use among women with POF.

## RESUMO

**CONTEXTO E OBJETIVO:**

Estudos com mulheres na pós-menopausa relatam aumento no risco de câncer de mama relacionado ao tipo e duração da terapia hormonal (TH) utilizada. Mulheres com falência ovariana prematura (FOP) representam desafio por necessitarem de TH prolongada. Pouco se conhece sobre ação da TH nas mamas dessas mulheres. Este estudo objetivou avaliar os efeitos de um tipo de TH sobre a densidade mamária de mulheres com FOP comparativamente à de mulheres pós-menopausa.

**TIPO DE ESTUDO E LOCAL:**

Estudo de corte transversal no Departamento de Tocoginecologia, Universidade Estadual de Campinas (Unicamp).

**MÉTODOS:**

Estudaram-se 31 mulheres com FOP e 31 mulheres na pós-menopausa, todas usando TH com estrogênio conjugado equino mais acetato de medroxiprogesterona, pareadas pelo tempo de utilização da TH. Todas realizaram mamografia, analisada por digitalização e por classificação de Wolfe, estratificada em duas categorias: não densa (padrão N1 e P1) e densa (P2 e Dy).

**RESULTADOS:**

Não houve diferença significativa entre a densidade mamária dos grupos analisadas por digitalização ou classificação de Wolfe. Pela digitalização, calculou-se densidade mamária média em 24.1% ± 14.6 e 18.1% ± 17.2 nas com FOP e pós-menopausa, respectivamente (P = 0,15); pela classificação de Wolfe identificou-se mamas densas em 51,6% e 29,0%, respectivamente (P = 0,171).

**CONCLUSÃO:**

Não se observou diferença na densidade mamária de mulheres com FOP comparativamente à de mulheres na pós-menopausa utilizando TH pelo mesmo período de tempo. Estes resultados podem auxiliar na aderência à TH de pacientes com FOP.

## INTRODUCTION

Studies carried out on postmenopausal women have shown increased risk of breast cancer relating to the type of hormone therapy (HT) used and the duration of its use.^1-7^ It is still a matter for debate whether HT causes a reduction in the sensitivity and specificity of mammographic screening as a result of the increase in breast density.^8-10^ The absolute risk of developing breast cancer, for a postmenopausal woman using estrogen-progestin HT, is individually very low (an increase of eight cases per 10,000 women annually). However, the cumulative effect has greater repercussions and is not considered insignificant in the case of prolonged use.^11^

Women with premature ovarian failure (POF) represent a challenge, since they require prolonged hormone therapy in view of their early loss of gonad function. However, little is known about the impact of prolonged HT use on these women's breasts.^11^ Concern about breast cancer is one of the most frequent causes of discontinuation of HT.^12^

Recently, postmenopausal changes in breast density, as evaluated by mammography, have been considered to be a strong marker for the risk of breast cancer. Breast density has been shown to reveal information on the exposure to endogenous and exogenous hormones that affect the environment in which cancer originates and develops.^10^ Various studies have shown that women with denser breasts have a two to six-fold higher risk of developing cancer, compared with women with less dense breasts.^13-17^

Much information linking HT and breast density in postmenopausal women has been published in the literature.^7,18-23^ However, to the best of our knowledge, no papers evaluating the breast density of women with POF have been published, even though these women are frequently treated with HT in the same way as postmenopausal women.

## OBJECTIVE

Considering the scientific evidence, the questions regarding the effect of estrogen-progestin hormone therapy on postmenopausal breast density and the lack of information on women with premature ovarian failure regarding this subject, a study was carried out to compare breast densities between women with POF who were using estrogen-progestin HT and postmenopausal women using the same type of HT for similar lengths of time.

## MATERIAL AND METHODS

This cross-sectional pilot study evaluated 31 women between 30 and 40 years of age with a diagnosis of POF shown by secondary amenorrhea with hypergonadotropic hypoestrogenism, with follicle-stimulating hormone (FSH) > 40 mIU/ml at two different times.^24^ These women were receiving care at the gynecological endocrinology outpatient clinic of the Department of Obstetrics and Gynecology, Universidade Estadual de Campinas (Unicamp), and they had been using an estrogen-progestin HT regimen composed of 0.625 mg of conjugated equine estrogen (CEE) combined with medroxyprogesterone acetate (MPA), cyclically or continuously, for at least 12 months. Women who had some form of pathological condition and/or had undergone previous breast surgery, women who smoked more than 20 cigarettes/day and those with body mass index (BMI) > 30 kg/m^2^ were excluded from the study.

This group was compared with a control group of 31 postmenopausal women who were using the same hormone therapy as a cyclic or continuous regimen. The control group women were matched with the women in the study group according to duration of hormone use (± 11 months). They were selected from the menopause clinic of the Department of Obstetrics and Gynecology, Unicamp. Women over 50 years of age for whom the menopause had been diagnosed at least 12 months previously were eligible for inclusion in the study. The exclusion criteria were identical to those of the study group.

All the women in both groups underwent mammography, and the data were analyzed and compared using both the technique of mammographic digitization^25^ and Wolfe's classification.^26^ However, the patients were stratified into only two groups: non-dense breasts (N1 and P1 patterns) and dense breasts (P2 and Dy patterns).

The study protocol was approved by the Institutional Review Board of the Department of Obstetrics and Gynecology, Unicamp, and by the Ethics Committee of the School of Medicine, Unicamp.

Mammography was carried out using a high-resolution scanner (CGR Senographe 500T, GE Medical Systems) with a Kodak RPX-OMAT processor. Kodak diagnostic films were used, and the left mid-lateral oblique incidence was used for digitization.

The mammogram films were placed on a negatoscope-type apparatus and covered by a sheet of transparent tracing paper. The outlines of the images corresponding to the fibroglandular and fatty portions were sketched by a specialist in mammography. Areas with the same density as the pectoralis major muscle were considered to be fibroglandular, while the remainder was considered to be fatty tissue. The drawings were digitized using a Hewlett Packard scanner and an IBM 486 desktop computer (DX4, 8 RAM, 540 HD). Digitization fragmented the figure into small areas referred to as pixels (picture elements). In the computer, the images were opened using an image editing software program (Paintbrush, Microsoft), in which the fatty areas were colored light grey and the fibroglandular areas were colored black. A numerical value of 250 was attributed to the light grey areas and zero to the black areas. The images were evaluated using the Mathlab4 software program by a specialist in physics, to quantify the percentage of glandular tissue in relation to the total volume of the breast, thus resulting in the dependent variable of breast density.

Evaluation of breast density was also carried out in accordance with Wolfe's classification. However, it was subdivided into only two categories: non-dense (N1 and P1 patterns, i.e. fibroglandular tissue accounting for < 25% of the breast volume in the left mid-lateral oblique incidence) and dense (P2 and Dy patterns, i.e. fibroglandular tissue accounting for ≥ 25% of the breast in the left mid-lateral oblique incidence). The specialist in mammography and the physicist who performed the analyses were blinded with regard to the identities of the groups.

Means and standard deviations were calculated to analyze the variables of age, parity and BMI. For the comparison of breast density between groups (digitization technique), Student's t-test for independent groups was used after performing the Kolmogorov-Smirnov test for normality. For analysis on Wolfe's classification, stratified into two categories, proportions were compared using the chi-square test in contingency tables.^27^ The significance level was established at 5%. The software used for the statistical analyses was the Statistical Package for the Social Sciences (SPSS), version 15.0 for Windows 2006.

## RESULTS

The mean age of the women with POF was 36.9 ± 2.9 years and the mean age of the postmenopausal women was 58.4 ± 5.1 years (variable approximately normally distributed in both groups: P = 0.388 and P = 0.652 respectively). The two groups were paired according to the duration of hormone therapy use (± 11 months). Hormone therapy (CEE + MPA) had been used for a mean of 50.3 ± 39.0 months by women in the POF group and for a mean of 50.1 ± 38.4 months by the women in the postmenopausal control group (variable also approximately normally distributed in both groups: P = 0.386 and P = 0.403 respectively). There was no statistically significant difference in duration of HT use between the two groups (P = 0.98). The mean time since diagnosis of POF was 85.9 ± 44.4 months, whereas the mean time since diagnosis of menopause was 117.7 ± 57.6 months (this variable also being approximately normally distributed in both groups: P = 0.870 and P = 0.680 respectively).

With regard to the other variables evaluated, the women with POF had had fewer pregnancies than had the postmenopausal women (1.5 ± 1.7 and 3.7 ± 2.6 pregnancies, respectively, P < 0.001; variable approximately normally distributed in both groups: P = 0.290 and P = 0.192 respectively). Fewer of the women with POF had breastfed than had the women in the control group (54.8% of the women with POF and 81.7% of the control group, P = 0.005).

Although women with BMI > 30 kg/m^2^ had been excluded from enrollment in the study, the difference in BMI between the group with POF (24.1 ± 3.2) and the control group (25.8 ± 3.3) was statistically significant (P = 0.04). BMI was approximately normally distributed in both groups (P = 0.809 and P = 0.480 respectively).

The percentage of breast density analyzed using mammographic digitization, a variable that was approximately normally distributed in both groups (P = 0.976 and P = 0.268 respectively), was 24.1 ± 14.6% in the POF group and 18.1 ± 17.2% in the control group. This difference was not statistically significant (P = 0.15). Wolfe's classification, stratified into two subgroups (non-dense: fibroglandular tissue occupying an area < 25% of the breast; and dense: fibroglandular tissue occupying an area ≥ 25% of the breast), also failed to detect any statistically significant difference in breast density between the two groups, although 51.6% of the women with POF had dense breasts, compared with only 29% of the postmenopausal group (P = 0.171) (Table 1).

**Table 1.** Breast density of women with premature ovarian failure (POF) and postmenopausal women (n = 31 in each group) analyzed according to digitization and Wolfe's classification

	Breast density according to mammographic digitization				
	POF		Postmenopausal (controls)		
% Breast density	Mean	SD	Mean	SD	P-value
	24.1	14.6	18.1	17.2	0.15*
	Breast density according to Wolfe's classification				
% non-dense	48.4		71.0		0.171†
% dense	51.6		29.0		0.171†

P-value < 0.05;

* Student's t test;

† Chi-square test (χ^2^).

SD = standard deviation.

## DISCUSSION

No difference in breast density was found between the women with POF and the postmenopausal women using hormone therapy with conjugated equine estrogens and medroxyprogesterone acetate for similar periods of time, either when analyzed objectively using digitization or subjectively in accordance with Wolfe's classification. However, in both groups, the percentage of fibroglandular tissue in relation to the total area of the breast was low (24.1 ± 14.6% and 18.1 ± 17.2%, respectively).

In the POF group, which was composed of younger women (mean age 36.9 ± 2.9 years), the mean time elapsed since gonad failure was shorter than in the control group. Other factors that could have protectively contributed towards the reduction in breast density were in fact less frequent in the POF group, in which the women had had fewer pregnancies and fewer women had breastfed. Despite these characteristics, breast density in the study group was no different from that of the control group.

This study does not enable conclusions to be reached regarding the causes of this finding, but one hypothesis may be that the hypoestrogenism following gonad failure, which would cause regression of fibroglandular tissue and its progressive replacement by fatty tissue,^18,20^ may have greater repercussions in reducing breast density when present at a younger age.

One concern regarding increased breast density resulting from estrogen-progestin replacement therapy relates to impaired mammographic sensitivity and specificity. This would result in a higher number of false-positive results, since the dense glandular tissue tends to make identification of tumorous masses more difficult,^9,10^ thereby compromising the early diagnosis of breast cancer.^8^

Hormone therapy is known to increase the density of the breast parenchyma. However, this does not occur in the majority of women,^18,20,22,28^ and this stimulus is also known to vary according to the type of hormone therapy used. In view of these factors, the groups were matched for duration of HT use and for the type of hormone used (CEE + MPA), Nonetheless, matching for exclusively cyclic or continuous use was not possible, since younger women with POF often want to menstruate, whereas postmenopausal women prefer continuous regimes in order to avoid bleeding.

Although age has been shown to have an inverse correlation with breast density,^29^ this association was not found in the study group or in the control group of postmenopausal women. Although the women in the study group were younger, their breast density was similar to that of the older postmenopausal women.

No significant difference in breast density was found between the two groups in this study, despite the fact that the groups had very different characteristics. This latter point has encouraged us to proceed with designing a new, prospective study involving data correlation that would enable greater precision of control over the variables that affect each woman participating in the study.

Finally, based on the results from this pilot study, and considering the likelihood of type I error as 0.05 (alpha = 0.05) and a test power (type II error) of 80% (1 - beta = 0.80), we recommend that for further studies, a sample size of at least 92 patients in each group should be used.

It is important to take into account the fact that the diagnosis of POF is established at a rate of about one case for every 1,000 women. This ratio may make it hard to achieve bigger samples than the present one.

Although the authors are aware of the limitations of the present sample size, this was a pilot study. Hence, the findings described here are important because this is the first paper reporting on the effects of estrogen-progestin HT on the breast density of women with POF compared to that of postmenopausal women using the same type of HT for similar periods of time.

The patients enrolled in this study are being followed up for prospective evaluation proposals.

Finally, it should be stressed that the question of hormone treatment remains open. New studies would be necessary, with bigger sample sizes if possible, in order to answer women's concerns and thus promote better compliance with treatment.

## CONCLUSIONS

There was no difference in breast density between the women with POF and the postmenopausal women who had used the same HT during similar periods.
